# Supplementary figures and images for: A kinesin-3 recruitment complex facilitates axonal sorting of enveloped alpha herpesvirus capsids
Source: PLoS Pathog. 2020 Jan 29;16(1):e1007985. doi: 10.1371/journal.ppat.1007985 (PMC7010296; doi:10.1371/journal.ppat.1007985)

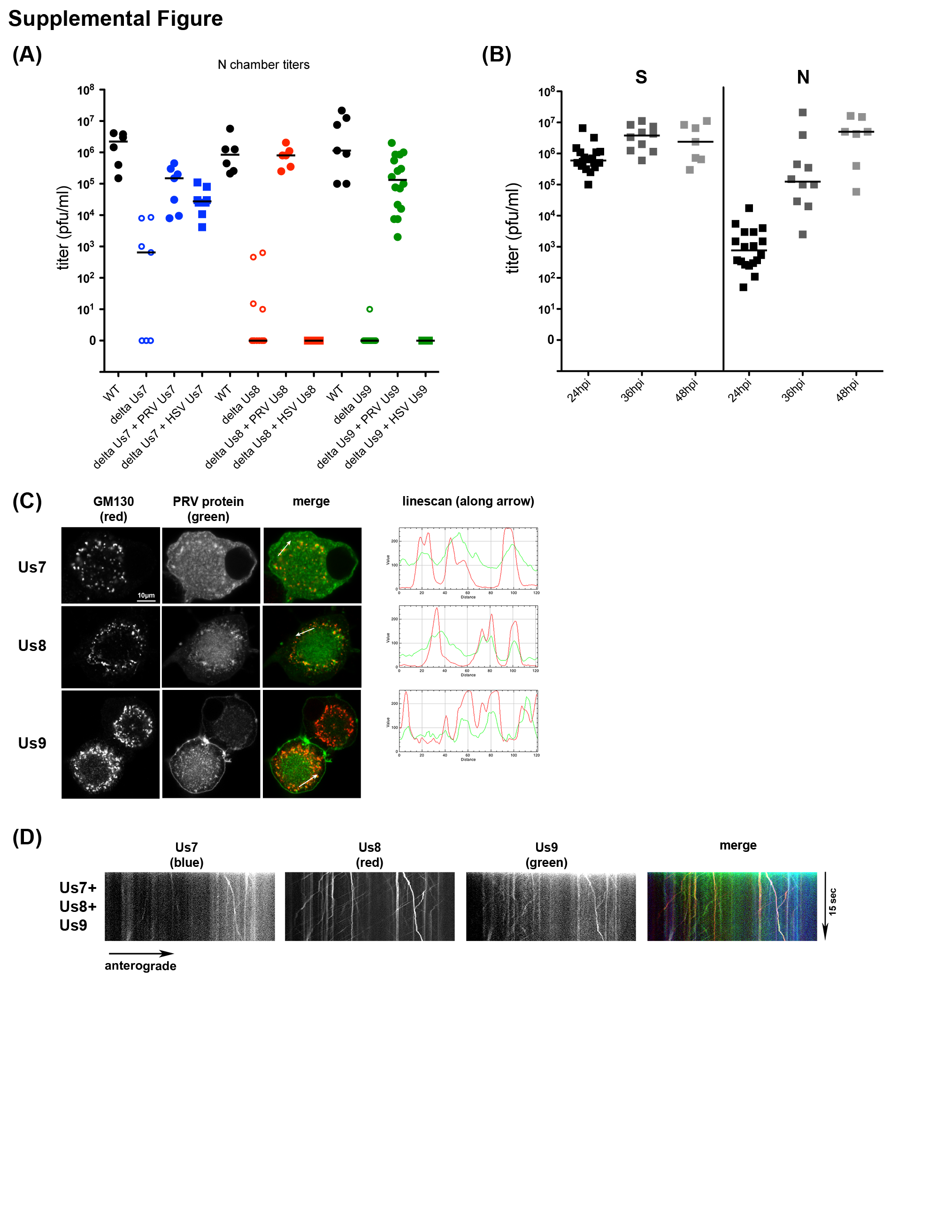

Supplement: S1 Fig — (A) Titers of PRV single Us7, Us8, or Us9 gene deletion mutants 24 hpi and rescue by adenovirus transduction with according PRV or HSV-1 gene produce. Effects on anterograde spread is indicated by N compartment titers. Each data point represents one chamber; horizontal bars indicate median values for each condition. Only HSV-1 Us7 is able to rescue PRV ΔUs7 virus, whereas HSV-1 Us8 and Us9 fail to rescue PRV ΔUs8 or ΔUs9, respectively. (B) Titers of HSV-1 wild-type infected anterograde spread assays at 24h, 36h, and 48h post-infection. Titers left and right of the solid vertical line represent S and N compartment titers, which indicate replication and anterograde spread, respectively. Each data point represents one chamber; horizontal bars indicate median values for each condition. Compared to PRV (Fig 1), HSV-1 has an up to 24h delayed anterograde spread phenotype. (C) Confocal imaging of SCG neuronal cell soma after transduction with indicated PRV proteins identical to Fig 2C. Linescans show colocalization of Golgi marker GM130 with PRV Us7, Us8, and Us9. (D) TIRF microscopy of live SCG axons grown in compartmentalized cultures. Cells were transduced with HSV-1 proteins Us7, Us8, and Us9 and imaged at ~12frames/s in three-color mode. Axonal co-transport of Us7-9 was observed without HSV-1 infection. (TIF) [file ppat.1007985.s001.tif]
